# Development and validation of a new multivariable prediction model to estimate risk of abnormal vault

**DOI:** 10.1186/s12886-023-02956-8

**Published:** 2023-05-10

**Authors:** Jing Yang, Zongyin Zou, Minhui Wu, Runzhang He, Yating Nong, Hui Li, Sheng Zhou

**Affiliations:** State Key Laboratory of Ophthalmology, Zhongshan Ophthalmic Center, Sun Yat-sen University, Guangzhou, People’s Republic of China

**Keywords:** Implantable Collamer Lens, Vault, Prediction model

## Abstract

**Purpose:**

To develop and validate a new multivariable prediction model to estimate risk of abnormal vault after EVO Implantable Collamer Lens (EVO-ICL) implantation using the preoperative parameters.

**Methods:**

This retrospective study comprised 282 eyes of 143patients who underwent EVO-ICL surgery between May 2021 and April 2022. We measured preoperative parameters before surgery and vaults in 1 week after the operation using swept-source optical coherence tomography (SS-OCT). Risk factors for abnormal vault were determined by univariate and multivariate logistic regression analyses, and a nomogram was developed to forecast the risk of abnormal vault after EVO-ICL implantation. We assessed the performance of nomogram in terms of discrimination and calibration, including concordance index (C-index), receiver operating characteristic curve (ROC), area under the ROC curve (AUC), and decision curve analysis (DCA). Bootstrap resampling was used as an internal verification method.

**Results:**

The logistic regression analysis revealed the independent risk factors for abnormal vault were white-to-white(WTW), anterior chamber angle(ACA), pupil size, and ICL-size, all of them were used to establish a nomogram based on multivariate logistic regression to predict the risk of abnormal vault. The C-indexes and AUC were 0.669 (95%CI, 0.605, 0.733). The calibration curves of the nomogram showed relatively small bias from the reference line, implicating an acceptable degree of confidence. The DCA indicates the potential clinical significance of the nomogram.

**Conclusions:**

We developed a new multivariable prediction model to estimate risk of abnormal vault. The model shows good prediction effect and can provide assistance for clinical decision of ICL size.

## Introduction

The EVO Implantable Collamer Lens (ICL), a type of posterior chamber phakic intraocular lens, is widely used for correction of refractive errors. After approximately two decades of conduction in clinical practice, ICL has been shown to be a safe and effective means to correct not only high myopia, but also low-to-moderate myopia, astigmatism, and hyperopia [1–3].

One of the vital post-operative parameters in classifying the surgery’s success is vault, the distance between the anterior crystalline lens surface and posterior ICL surface. A low vault (< 250 μm) was associated with the development of lens opacity [4–6]. On the other hand, a high vault (> 750 μm) may increase the risk of angle closure, pupillary block or pigment dispersion glaucoma [5, 7, 8],other studies have reported that vault was the most significant factor for changes in endothelial cell density (ECD), excessively high vault values increased the risk of ECD loss and the occurrence of glaucoma [9, 10].

Unfortunately, with nomogram and the two prediction formulas recommended by ICL manufacturer, prediction of vault and determination of ICL size remains an unresolved problem [11–13]. Previous researches have been focused on establishing formulas [14], developing new algorithms [15] and artificial intelligence according to the preoperative parameters [16] to predict the vault more accurately. However, due to the complication of anterior segment biometrics, the preoperative parameters identified by previous studies to calculate vault remains controversial.

Theoretically, the vault is related to parameters such as anterior chamber depth (ACD) horizontal angle-to-angle diameter (ATA) ,Crystalline lens rise (CLR) and white- to -white (WTW) and the size of ICL. In practice, there are only four sizes of ICL: 12.1 mm, 12.6 mm, 13.2 mm, and 13.7 mm for surgeons to choose referring to manufacturer’s prediction formulas, which suggests that the relationship between vault and preoperative parameters may not be sufficiently explained using multiple linear regression. In addition, the optimal vault is between 250 and 750 μm, a review showed that no mean value for each of the individual studies was found to be outside of this range (values varied from 340 to 637 μm) [8]. Excessively low or high vault should be defined as abnormal vault, but there is no evidence that in optimal range (250 to 750 μm), lower or higher vault is related to postoperative complications, indicating that it is unnecessary to calculate the precise value of vault. Therefore, we innovatively divided the vaults into two categories: optimal vault (250 to 750 μm) and abnormal vault (< 250 or > 750 μm) instead of precise value to explore the relation between vault and preoperative parameters.

The aim of our research is to develop and validate a new multivariable prediction model to estimate risk of abnormal vault after EVO-ICL implantation using the preoperative parameters.

## Methods

### Inclusion and exclusion criteria

**Fig. 1:**
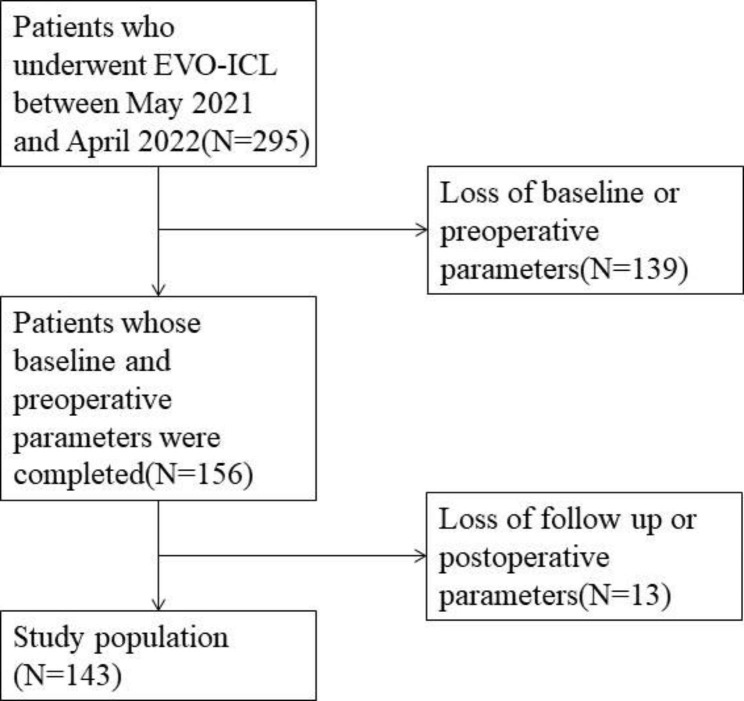
Flow diagram of the patients’ enrollment process

This retrospective study included 143 patients who underwent EVO-ICL surgery to treat myopia at Zhongshan Ophthalmic Center, China between May 2021 and April 2022 by 2 senior surgeons. The enrollment process of patients is shown in Fig. 1. The inclusion criteria were (1)corneal ECD > 2000/mm^2^.(2)ACD > 2.8 mm, and (3)completed EVO-ICL surgery and follow-up in this hospital. The exclusion criteria were:(1) patients who were diagnosed with other ocular conditions, such as cataract, glaucoma and corneal dystrophy;(2) patients who had systemic diseases such as diabetes, autoimmune disease and other diseases that can influence the parameters after surgery; (3)patients whose baseline and preoperative parameters was incomplete, and (4) patients lost to follow up or have incomplete postoperative parameters. The median follow-up time of patients was 1 months. According to these criteria, 282 eyes in 143 patients were selected .ICL sizes were selected by Online Calculation and Ordering System (OCOS) provided by STAAR Surgical.

### Preoperative and postoperative protocol

The parameters were defined as follows: white- to-white (WTW) was defined as the distance between nasal and temporal limbus points between the white sclera. Anterior chamber depth (ACD) can be regarded as the distance between the anterior surface of the crystalline lens and posterior surface of cornea. The horizontal angle-to-angle diameter (ATA) was the distance between the angle recesses on the nasal and temporal sides. Crystalline lens rise (CLR) was defined as the anteroposterior distance between the anterior crystalline lens surface and the angle recess to angle recess line. The postoperative vault was the distance between the posterior surface of the ICL and the apex of the crystalline lens .Anterior chamber angle (ACA) was the angle between peripheral cornea and the root of iris .A swept-source optical coherence tomography instrument (SS-OCT, VG200D, SVision Imaging, Ltd., China)was used to measure the ACD ,ATA ,CLR ,pupil size, postoperative vault ,and temporal ACA .The SS-OCT scans were performed along the horizontal meridian using a single-scan centered on the pupil by 1 operator .AL (Axial length) was measured by IOLmaster. WTW was measured by a caliper under slit-lamp (The caliper’s lowest unit of measurement was 1 mm). Other parameters were included age, and the size of the ICL selected by Online Calculation and Ordering System (OCOS). All measurements were taken under the same mesopic conditions.

### Surgical protocol

Before surgery compound tropicamide eye drops (Zhuo bian™; Shenyang Xing Qi Eye Drops Medicine Co., Ltd; China ) were used for mydriasis. Surface anesthesia were performed by Proparacaine Hydrochloride (Alcaine™; s.a.ALCON-CONUVERUR n.v;Belgium). The axis for astigmatism was marked before surgery. The operation was performed aseptically. The main 3.2 mm incision was made at the steepest meridian of the cornea, then the ICL was injected into anterior chamber using manufacturer injector cartridge (STAAR Surgical Co.) after the viscoelastic material (Singclean™; Hangzhou Singclean Medicine Products Co., Ltd; China) had been placed into the anterior chamber ,and moved to the posterior chamber through pupil .After correcting the position of lens, viscoelastic material was washed out using a balanced saline solution. After surgery, antibiotic (0.3% Ofloxacin, Tarivid™; Santen Pharmaceutic Co., Ltd; China), drugs were applied topically 4 times a day for 4 weeks.

### Statistical analysis

IBM SPSS Statistics 25 (SPSS Inc., Somers, NY, USA) and .R software version 4.2.1 (The R Foundation for Statistical Computing, Vienna, Austria. http://www.r-project.org) was used to run the statistical analysis. In this study, the R packages including “rms”, “Hmisc”, “pROC”, “rmda” and “Decision Curve” were used to build the nomogram, plot the area under the ROC curve (AUC), conduct decision curve analysis (DCA) and calibration curve. P < 0.05 was considered statistically significant. The nomogram was based on the multivariate logistic regression analysis. The sample size is enough to drawn a safe conclusion according to the standard that a fitted regression model is likely to be reliable when the number of predictors (or candidate predictors if using variable selection) is less than m/15, where m is the “limiting sample size”. [17, 18]

## Results

### Patient characteristics

In our research, a total of 282 eyes were included in our study. Descriptive statistics for the preoperative and post-operative data of our study population are presented in Table 1.

**Table 1:** Characteristics of the Study Population

Parameter	Value
Age(years)	27.63 ± 6.46(17.00to46.00)
Sex(M/F)	45/98
Eye(OD/OS)	139/143
AL(mm)	27.83 ± 2.12(18.19to35.44)
WTW(mm)	11.79 ± 0.51(10.80to13.50)
ICL-sphere(D)	-12.33 ± 3.78(-18.00to-3.00)
ICL-cylinder(D)	-1.08 ± 1.30(-5.00to0)
ICL-SE(D)	-12.87 ± 4.06(-20.50to-3.00)
ICL-size(mm)	
12.1	80(28.4%)
12.6	137(48.6%)
13.2	62(22.0%)
13.7	3(1.1%)
ACD(mm)	3.25 ± 0.23(2.10to3.76)
ATA(mm)	11.76 ± 0.99(1.16to12.94)
CLR(µm)	2.98 ± 217.33(-480.80to1115.43)
ACA(°)	52.16 ± 14.36(22.00to94.10)
Pupil size(mm)	4.50 ± 0.90(2.19to6.57)
Vault(µm)	605.75 ± 295.54(21.30to1670.70)
Optimal Vault	103(36.5%)
Abnormal Vault	179(63.5%)

Abbreviations: AL, axial length; WTW, white- to-white; ACD, anterior chamber depth; ATA, angle-to-angle diameter; CLR, crystalline lens rise; ACA, anterior chamber angle

### Identification of the risk factors for abnormal vault

We performed univariate logistic regression analysis to explore the risk factors of abnormal vault. ACA(OR, 0.978; 95%CI, 0.961, 0.995; p = 0.015); Pupil size (OR, 1.445; 95%CI, 1.097, 1.919; p = 0.010)ICL-size13.2(OR, 3.200; 95%CI, 1.588, 6.600; p = 0.001) and WTW(OR, 2.357; 95%CI, 1.435, 3.955; p = 0.001) were statistically significant using univariate logistic regression analysis (Table 2).

**Table 2:** Univariate logistic regression analyses of risk factors for abnormal vault

term	Correlation coefficient	OR	p
Pupil size*	0.368	1.445 [1.097, 1.919]	0.01
ACA*	-0.022	0.978 [0.961, 0.995]	0.015
CLR	0.001	1.001 [0.999, 1.002]	0.308
ATA	0.002	1.002 [0.781, 1.350]	0.986
ACD	0.587	1.799 [0.618, 5.444]	0.288
ICLSE	-0.018	0.982 [0.924, 1.042]	0.546
ICLcylinder	-0.109	0.896 [0.745, 1.079]	0.244
ICLsphere	-0.007	0.993 [0.961, 1.025]	0.654
ICLsize12.6	0.513	1.670 [0.913, 3.137]	0.102
ICLsize13.2*	1.163	3.200 [1.588, 6.600]	0.001
ICLsize13.7	1.792	6.000 [0.547, 133.232]	0.152
Cylinder	-0.161	0.852 [0.704, 1.029]	0.096
Sphere	0.002	1.002 [0.946, 1.061]	0.958
WTW*	0.857	2.357 [1.435, 3.955]	0.001
AL	0.037	1.037 [0.925, 1.164]	0.529
EyeOS	-0.137	0.872 [0.536, 1.417]	0.581
Age	-0.029	0.971 [0.934, 1.009]	0.141
SexM	0.245	1.277 [0.759, 2.139]	0.353

Abbreviations: AL, axial length; WTW, white- to-white; ACD, anterior chamber depth; ATA, angle-to-angle diameter; CLR, crystalline lens rise; ACA, anterior chamber angle.*There was statistical difference

### Construction of predictive model for abnormal vault

**Fig. 2:**
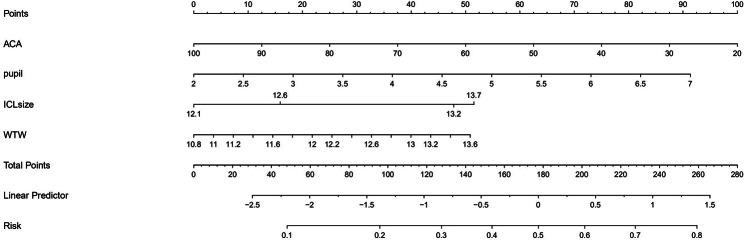
The nomogram to estimate risk of abnormal vault Abbreviations: WTW, white- to-white; ACA, anterior chamber angle

Based on the univariate logistic regression analysis for abnormal vault, all the independent significant risk factors (ACA, Pupil size, ICL-size and WTW) were selected to perform the multivariate logistic regression analysis and build the predictive model to estimate risk of abnormal vault. The new multivariate prediction model is presented in the form of graphical calculator (nomogram), which was illustrated in Fig. 2.

 The nomogram transforms each one of the risk predictor into 0 to 100 points that is proportionally based on adjusted log odds. The points of each predictor should be added to derive the ‘‘total points’’, which are converted to the risk of abnormal vault. For individualized prediction, draw an upward vertical line to the “Points” bar to calculate total points corresponding to the patient’s characteristics. Then, draw a downward vertical line from the “Total Points” line based on the sum to calculate the linear predictor (inner product of weight vector and feature vector) and convert to the risk of abnormal vault.

### Validation of the new predictive model to estimate risk of abnormal vault

**Fig. 3:**
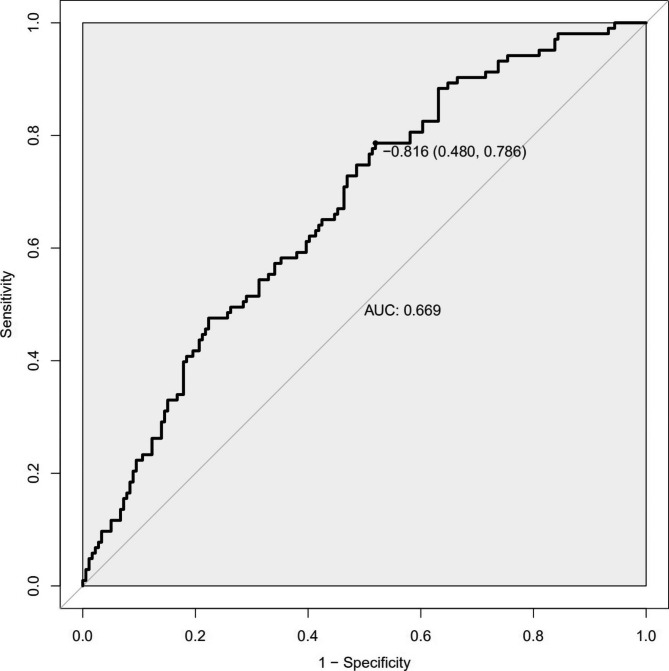
The ROC and AUC values to of nomogram. The sensitivity of the prediction model is plotted on the x-axis; the value of 1-specificity of the prediction model is plotted on the y-axis

In this study, C-index value and AUC were applied to evaluate the discrimination ability of the new predictive model; moreover, C-index value and AUC were adjusted through 1,000 bootstraps as internal validation to ensure that the nomogram had good effect in predicting abnormal vault. C-index value was 0.669(95%CI, 0.605, 0.733). The adjusted value of the C-index was 0.635, and as mentioned above, the AUC was the same as the C-index value (Fig. 3). According to the receiver operating characteristic curve (ROC), the point marked on Fig. 3 shows that when threshold is determined to be -0.816(linear predictor in nomogram), the sensitivity of the prediction model is 78.6%, and the specificity is 52.0%.

### Net benefit and predictive capacity of the new predictive model

**Fig. 4:**
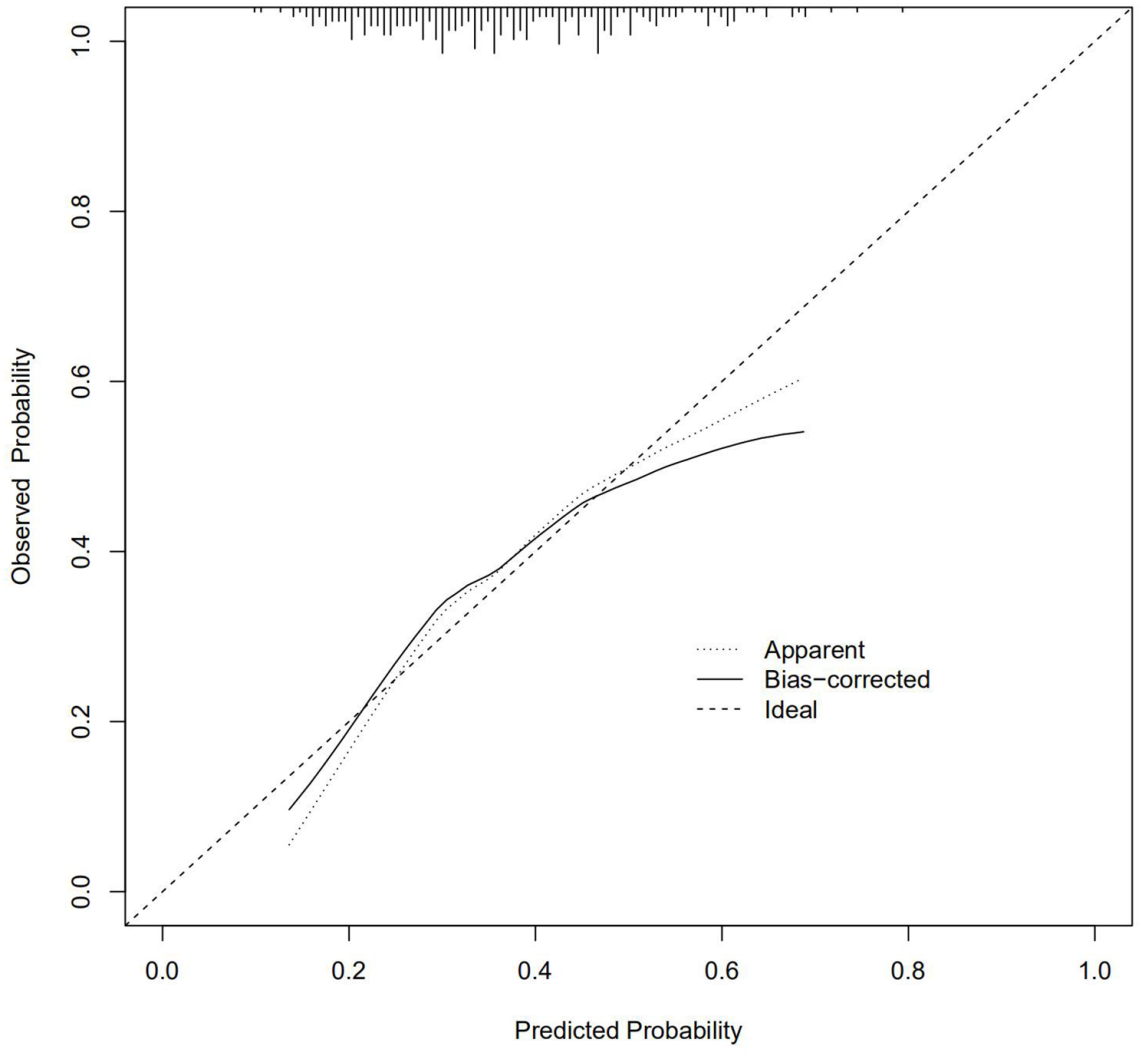
The calibration curve of predictive nomograms for predicting abnormal vault. Nomogram-predicted probability is plotted on the x-axis; actual probability is plotted on the y-axis. The distribution of the predicted probabilities of abnormal vault is shown at the top of the graph

 Furthermore, the calibration curves of our new model for predicting abnormal vault also used 1,000 bootstraps for internal validation. According to Fig. 4, the distance of each curve represents the difference between the nomogram estimated risk and the observed risk. The adjusted calibration curves showed that the deviation from the reference line are relatively small, indicating a good level of confidence.

**Fig. 5:**
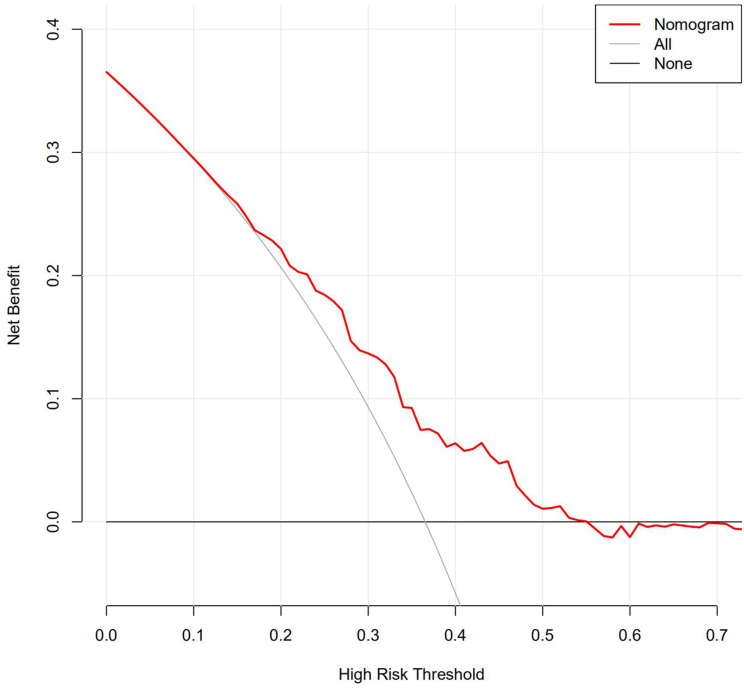
The benefit curve represented by the nomogram. The y‑axis indicates the overall net benefit, which is calculated by summing the benefits (true positive results) and subtracting the harms (false positive results).The x-axis indicates the threshold that used to decide whether it is high risk to have abnormal vault. All: Net benefit curve when all samples are abnormal vault; None: Net benefit curve when all samples are single metastasis

As one of the effective methods for evaluating the prediction of a new model, the decision curves analysis (DCA) implicates the usefulness of decision-making in clinical practice by calculating the clinical effects. The DCA curves for the predictive nomogram are presented in Fig. 5. As shown in Fig. 5, the DCA showed that the nomogram had relatively high overall net benefit.

Taken together, these results prove the value of our prediction model that has potential clinical significance and a good performance to predict abnormal vault.

## Discussion

As the most vital postoperative parameter after EVO-ICL surgery, vault has drawn wide concern of researchers. Prior studies developed a variety of formulas and algorithms to explore the relationship between preoperative parameters and vault, but the conclusions and factors that significantly affect the vault were still not clear. For instance, NK and KS formula [11–13] have been commercially available for predicting the ICL vault, but the factors they used to build the formulas are completely different: according to NK formula, the predicted ICL vault = 0.5 + 1.1 × (implanted ICL size − optimal ICL size using the NK formula) [11], while in KS formula, predicted ICL vault (µm) = 660.9 × (ICL size (mm) − ATA (mm)) + 86.6 [12]. However, the traditional tool to recommend the optimal ICL size, the manufacturer’s nomogram was based on WTW and ACD. A study aiming to compare the achieved vault using formulas above showed that the achieved vault was significantly smaller than the predicted vaults using the NK and KS formulas respectively, and the agreement rate of the recommended ICL size using the manufacturer’s nomogram, the NK formula, and the KS formula was 50.0% [19]. Many researches have been carried out to predict vault more precisely: Trancon et al. built a formula that explained 34% of the vault variance [14]; 36% found by Zheng et al. [20]; and Lee et al. reported a formula that explained37% of the vault variance [21], but there is still no general agreement about the parameters that should be identified as risk factors of abnormal vault.

The reason previous studies fail to build a wide-accepted model to predict vault effectively is complicated. In theory, the parameters that measure the size of anterior chamber may show a linaer correlation of the vault in the patients implanted with particular size of ICL. While the accuracy of parameters could be a challenge when trying to explore the factor that influence vault since it has been reported that biometric measurements such as WTW with different technologies showed large discrepancies with low correlation levels [22],and interexaminer variance in STS measurement should be considered [23]. In addition, researchers have found that the vault could be unpredictable due to immeasurable posterior chamber anatomic factors such as ciliary body [24]. Chen et al. reported eyes with an anteriorly positioned ciliary body were associated with a higher rate of excessive vault [25]. Other researchers categorized the iris shape into three groups, and they found concave shape iris had a higher risk of low vault and convex shape iris were more likely to demonstrate high vault in eyes with thick lens [26, 27].Therefore, a new tool that based on different perspective need to be developed.

In this retrospective study, we innovatively divided the vaults into two categories: optimal vault (250 to 750 μm) and abnormal vault (< 250 or > 750 μm). Further, we identified independent risk factors of abnormal vault, including WTW, ACA, pupil size, and ICL-size. On the basis of these preoperative parameters, we established a nomogram to estimate risk of abnormal vault. The discrimination and calibration of the nomogram were proved, and this nomogram shows a good predictive effect. These results therefore need to be interpreted with caution since that the risk factors we found couldn’t be simply explained by traditional opinion such as there are linear interrelationship between preoperative parameters and precise value of vault. This new prediction model could contribute to our understanding of the reason why abnormal vault still exist after detailed calculation.

WTW is generally acknowledged as an indirect parameter to measure the size of posterior chamber. According to the nomogram we built, WTW is positively correlated with the risk of abnormal vault, but its points are relatively low, which indicates that after selecting optimal ICL size based on WTW, the influence of WTW has been partly diluted. Given the ICL haptic footplates are located on the ciliary sulcus, some researches took anatomic factors such as horizontal compression by the iris into consideration [21]. Therefore, the optimal ICL size should be adapted to the structure of posterior chamber, our study revealed that on condition that the optimal ICL has been selected, patients who have lager WTW along with lager size of ICL are more likely to have abnormal vaults after surgery.

In practice, there are only four types of ICL-size: 12.1 mm, 12.6 mm, 13.2 mm, and 13.7 mm, which may explained why the ICL-size was found to be a risk factor of abnormal vault. The bigger ICL we choose, it is more likely to achieve abnormal vault, which may support the theory that other confounding factors such as the friction and softness of the ciliary sulcus structure would make the vault unpredictable [21], and with larger size of ICL implanted, the influence of confounding factors is amplified. In the idealized model, horizontal compression generated by iris is the main factor that forms the vault. However, the iris also has vertical compression on the ICL, pushing the ICL close to the crystalline lens and inserting the ICL footplates into the ciliary sulcus, especially when the bigger size of ICL was chosen [21, 28], leading to the deviation of the predicted and actual vault and higher risk of abnormal vault.

In addition, the angle between peripheral cornea and the root of iris, ACA, was a preventive factor, bigger ACA suggested lower risk of abnormal vault according to the nomogram. Eyes with large ACA have flat iris. As is elaborated above, when the ICL lens was implanted behind the iris, more flat iris would generate greater vertical compression and enlarge the friction of haptic footplates against the iris, which causing more risk of unpredictable achieving vault.

Moreover, our predictive model included pupil size as a risk factor. The relationship between pupil and vault changes has been widely investigated, several studies have revealed that pupil constriction leads to decrease of vault under photopic conditions [29, 30]. It could be explained that as the pupil construct, the iris tension increase, pushing the ICL towards the crystalline, which leads to lower vault. Such theory are unsatisfactory because the changes of vault caused by pupil and iris movement are also influenced by other complicated factors. An example of these is the study carried out by Xiong et al. which suggested that an extremely low vault has a big rate of vault change, while an extremely high vault would constrict the posterior movement of pupil on mesopic condition. [31] On the other hand, the ciliary muscle contraction along with pupil construction makes ICL bend and adapt to the posterior surface of the iris, which could also cause unpredictable outcomes and eventually leads to abnormal vault. In our study, the pupil size was measured on mesopic condition, which partly represents the general tension of iris. A possible theory is that the bigger pupil is related to inclination of ciliary and iris transformation, which was also supported by another research that showed increasing pupil size is associated with vault change postoperatively from 1 day to 1 week [32].

There are some limitations in our research. First of all, for a multivariable prediction model, our research was on the basis of a relatively small number of cases, so we plan to conduct further study and enlarge the study population. Secondly, our follow-up time is relatively short, the vault we analyzed was measured in one week after surgery. Although it is reported that the vault was meanly change in one week after surgery [33, 34], it would be more convincible if we increase follow-up time and get more comprehensive data., which means the predictability may be imprecise with time goes by. What’s more, we used the data of two eyes of each patient so we could not avoid the effect of not independent eyes. The reasons we analysis both eyes of patients in this study is as follows: For one thing, the data volume is critical for the accuracy and clinical significance of the prediction model, so we decided to use the data from both eyes of patients to satisfy the data size required in our study. For another, the parameters and risk factors we defined in our study are relatively independent to each eye, which is supported by the fact that many patients achieve different vaults in both eyes with different size of ICL. We are planning to perform further research in the future that includes more patients’ independent eyes. Finally, due to the relatively strict standard we used to divide the vaults into two categories and individual differences, the rate of abnormal vault is high in our studies. But it won’t generate too much bias to our results since the data was reliable and analyzed carefully.

## Conclusion

We developed a new multivariable prediction model to estimate risk of abnormal vault. The model shows good prediction effect and can provide assistance for clinical decision of ICL size. Surgeons could select the size of ICL on the basis of nomogram to reduce the risk of abnormal vault and decrease the rates of ICL re-change after EVO-ICL surgery. Furthermore, the parameters we identified to build prediction model may shed a light on future researches that aim to explore the method to predict the vault more accurately.

## Data Availability

The datasets used and analyzed during the current study are available from the corresponding author on reasonable request.

## List of abbreviations

ICL: Implantable collamer lens
WTW: White-to-white
ACD: Anterior chamber depth
ATA: Angle-to-angle diameter
CLR: Crystalline lens rise
ACA: Anterior chamber angle
STS: Sulcus to sulcus
OCT: Optical coherence tomography
SD: Standard deviation
AUC: Area under the curve
DCA: Decision curve analysis
